# Assessment of residual stumps 12 months after saphenectomy without high ligation of the saphenofemoral junction

**DOI:** 10.1590/1677-5449.210029

**Published:** 2021-07-05

**Authors:** Giovanna Golin Guarinello, Francisco Eduardo Coral, Jorge Rufino Ribas Timi, Sarah Folly Machado

**Affiliations:** 1 Hospital Santa Casa de Curitiba – HSCMC, Curitiba, PR, Brasil.; 2 Pontifícia Universidade Católica do Paraná – PUCPR, Curitiba, PR, Brasil.; 3 Universidade Federal do Paraná – UFPR, Curitiba, PR, Brasil.

**Keywords:** varicose veins, venous insufficiency, saphenous vein

## Abstract

**Background:**

Currently, the first-choice option recommended for varicose vein surgery is thermal ablation of the saphenous vein, but this procedure is not available on the Brazilian National Health Service (SUS - Sistema Único de Saúde). In an effort to improve results, surgical techniques have been developed to mimic the new technologies, without their high costs. The most prominent such method involves conventional saphenectomy, without ligation of tributaries.

**Objectives:**

To assess progression of the residual stump after saphenectomy without high ligation of the saphenofemoral junction but with stump invagination and to assess the behavior of anterior/posterior accessory veins.

**Methods:**

Prospective intervention study. A total of 52 limbs were treated with saphenectomy without high ligation of the saphenofemoral junction followed by invagination of the residual stump. Patients were assessed preoperatively and at 7 days, and 3, 6, and 12 months postoperatively using vascular ultrasonography with Doppler to analyze the length of the residual stump, the diameters of the residual stump and the anterior/posterior accessory vein, reflux in the accessory vein, and presence of neovascularization. Statistical analysis involved calculation of means, standard deviations, medians, minimum and maximum values, frequencies, and percentages, and Fisher’s test and the binomial test.

**Results:**

There was evidence of a significant time effect on residual stump diameter (p < 0.001) and length (p = 0.002), but the same was not observed with relation to diameter (p = 0.355) or reflux of the anterior accessory vein. Neovascularization was found in 7 (14.3%) limbs.

**Conclusions:**

After use of the technique described, the residual stump retracted, its diameter reduced over the 1 year postoperative period, and it did not transfer reflux to the accessory vein. Neovascularization rates were in line with the literature.

## INTRODUCTION

Lower limb varicose veins affect around 35-50% of the Brazilian population^1^^-^4 and are a common reason for seeking medical care. Although benign, they have a considerable impact on quality of life and significant socioeconomic implications, in terms of healthcare costs and days absent from work.^3^^,^^5^^,^^6^

From 60 to 80% of patients with varicose veins have reflux at the saphenofemoral junction (SFJ),^1^ and the European Society for Vascular Surgery (ESVS) chronic venous insufficiency (CVI) guidelines recommend surgical treatment rather than conservative management for uncomplicated varicose veins.^7^ For many years, the gold standard for surgical treatment of symptomatic CVI with great saphenous vein (GSV) insufficiency was conventional surgery with high ligation of SFJ tributaries, followed by removal of the GSV by stripping.^6^^,^^8^ However, studies suggest recurrence rates of 25-50% over 5 years.^9^

One of the main causes of recurrence is neovascularization, which occurs in the form of new dilated and tortuous veins that emerge where the SFJ has been manipulated.^6^^,^^10^^,^^11^ One hypotheses for this is endothelial exposure of the residual stump and methods to reduce this include invagination with non-absorbable sutures or interposition of an anatomic barrier, whether with the cribriform fascia or polytetrafluoroethylene (PTFE).^7^

The endovenous treatments that are now considered the first choice for treatment of GSV reflux^7^ by the SECV guidelines have questioned the principle of ligation of all vessels upstream of the SFJ, maintaining it in place above the pre-terminal valve.^11^^-^14 The results of these techniques demonstrated that the rate of medium-term SFJ reflux did not exceed 15%, with anterograde drainage of tributaries in the direction of the femoral vein in 85 to 100% of cases.^13^ Although conventional surgery and endovenous treatment have similar rates of varicose vein recurrence after 2 years, neovascularization at the SFJ appears to be more common among patients who undergo conventional surgery.^10^ Thus, techniques for endovenous treatment are conducted without high ligation of the SFJ, which may be an advantage, because it reduces neovascularization rates, since there is no endothelial exposure, and also preserves anterograde drainage from tributaries to the femoral vein.

Although endovenous techniques are less invasive and are associated with lower neovascularization rates, conventional surgery with high ligation of the SFJ is still widely employed.^8^^,^^14^ In Brazil, an average of 65,728 varicose vein surgeries per year were performed by the Brazilian National Health Service (SUS - Sistema Único de Saúde) from 2016 to 2019, considering both unilateral and bilateral operations,.^15^ Since neither the SUS nor private healthcare insurers cover endovenous techniques, conventional surgery is the surgical technique most used in Brazil. These data justify this study, with the objective of improving lower cost techniques through application of the hemodynamic principles that underlie endovenous management.

Maintenance of the SFJ may be preferable to conventional surgery because it is less invasive and is associated with lower risk of local inflammatory reactions. Use of an approach that involves reduced manipulation and no SFJ dissection appears to stimulate neovascularization less while also preserving inguinal venous drainage.^13^ The advantages of GSV surgery without high ligation include the lower cost of the procedure^8^ compared to endovenous surgery, coverage by private healthcare insurers and SUS (since the same materials and techniques are used as for the technique with high ligation), and possibly lower rates of relapse than conventional surgery.^11^ Invagination of the residual stump, in combination with the above technique, is intended to minimize the risk of neovascularization further still.

## OBJECTIVES

To assess the behavior of the residual saphenous stump after saphenectomy of the GSV without high ligation of the SFJ, but with invagination of the residual stump and also to assess the behavior of the anterior/posterior accessory veins.

## METHODS

The is a prospective intervention study that was approved by the Research Ethics Committee (CAAE: 79980117.1.0000.0020, ruling number 2.824.708). A total of 46 patients were analyzed who underwent surgical treatment for lower limb varicose veins with technique described below by the same team of surgeons. Patients who had been prescribed surgery and met inclusion and exclusion criteria were selected from a Lymphedema and Angiodysplasia Clinic run by the SUS. Patients were recruited by consecutive sampling from January 2018 to July 2019.

Inclusion criteria were age greater than 18 years, presence of reflux at the SFJ (> 0.5 seconds during vascular ultrasonography [US] examination) and signature of a free and informed consent form. Exclusion criteria were: reflux at the anterior and/or posterior accessory saphenous, body mass index > 35, and prior history of treatment of the SFJ in the same limb.

The patients selected were assessed preoperatively with patient history, physical examination, and vascular US with Doppler examination of the limb to be operated on. With the aim of confirming the presence of reflux at the SFJ, ruling out its presence in the anterior/posterior accessory saphenous vein, and measuring the diameter of the junction and the accessory vein(s), another US vascular examination with Doppler was conducted in the operating room, with patient in orthostatic position, prior to initiation of anesthesia by a physician with experience in vascular US.

**Figure 1:**
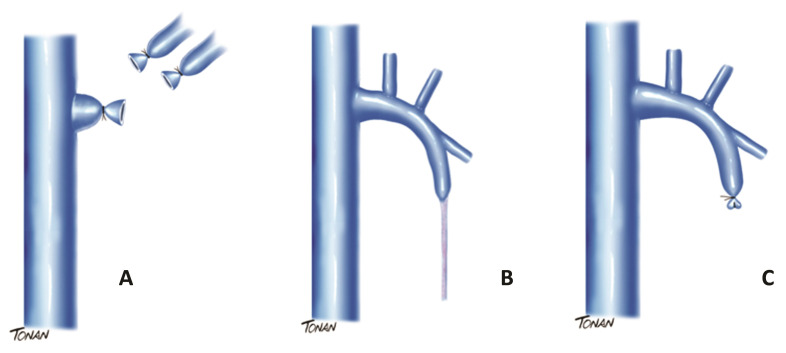
Comparison between surgical techniques for treatment of the great saphenous vein. (A) Saphenectomy with hig ligation; (B) endovenous treatment; (C) Without ligh ligation, but with stump invagination.

Postoperative assessments were conducted at 7 days, 3 months, 6 months, and 1 year, using vascular US with Doppler (Mindray M5 model), measuring the following parameters: diameter and length of the residual stump; presence of reflux in the anterior/posterior accessory saphenous vein; and presence of neovascularization. All of the postoperative imaging exams were performed in orthostatic position and by the same examiners as the preoperative exams. Neovascularization was defined as new tortuous veins and veins with reflux close to the SFJ. Figure 1 illustrates the technique employed in a simplified manner, comparing it with conventional surgery and the endovenous technique.

**Figure 2:**
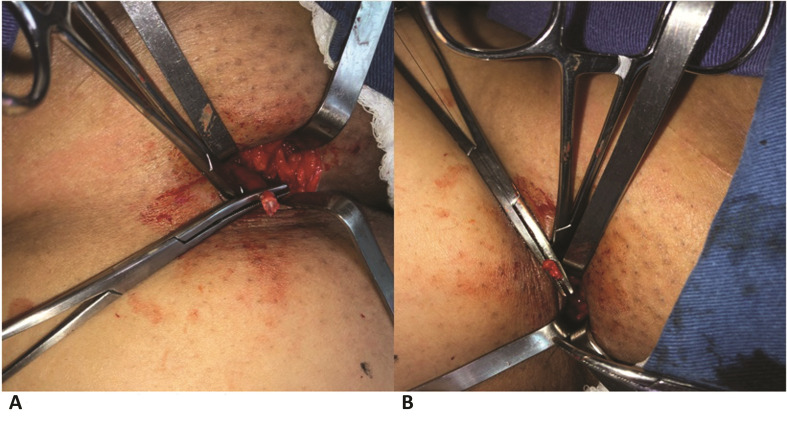
Surgical technique - invagination of the residual stump. (A) Exposure of endothelium; (B) Stump invagination.

Surgery was initiated by making incision measuring approximately 3 cm in the inguinal fossa of the limb involved. Using a minimum of local manipulation, the GSV and its most distal tributary were located. After repairing it below the tributary, with the aid of two Kelly clamps, the vein was sectioned and proximal ligation was conducted with Vicryl 3-0®, at which point it was possible to observe endothelial exposure of the residual stump. The operation proceeded with invagination of the stump using continuous 5-0 monofilament nylon sutures. Depending the extent of the reflux, total or partial saphenectomy was then performed using the stripping technique. Additional varicose branches were also treated by vein stripping. After review of hemostasis, the skin was drawn closed with 5-0 nylon monofilament. Figure 2 shows invagination of the residual stump.

For statistical analysis, results for quantitative variables were expressed as means, standard deviations, medians, and minimum and maximum values. Categorical variables were expressed as frequencies and percentages and Fisher’s exact test was used to determine associations between two categorical variables.

The sample size was calculated on the basis of the results from the first 15 limbs analyzed in the study, considering a 5% significance level, 90% test power, and a standard deviation of 2 for the difference between two assessments. According to this calculation, 44 limbs would be needed to detect a 1 mm difference between two assessments with significance. This number was increased by six limbs to allow for possible losses to follow-up. The sample size was therefore defined as 50 limbs.

The assessment of residual stump behavior was based on an analysis of SFJ diameter and length over time. For each of the measurements, the null hypothesis that time had no effect on the measurement was tested against the alternative hypothesis that it did. Since it was considered that as a result of not performing high ligation of the SFJ, the residual stump might transfer reflux to the tributaries, causing recurrence of varicose veins, the anterior/posterior accessory vein was assessed, also by null hypothesis, for development of reflux and its diameter was monitored.

The effect of time on SFJ diameter, stump length, and accessory vein diameter were analyzed using a mixed-effects model, considering intercept and slope as random effects and time as a fixed effect. A binomial test was used to compare follow-up dates with the proportion of accessory veins in which reflux was identified. Statistical significance was indicated by p values < 0.05. Data were analyzed using IBM SPSS Statistics v.20.0. Armonk, NY: IBM Corp.

## RESULTS

**Figure 3:**
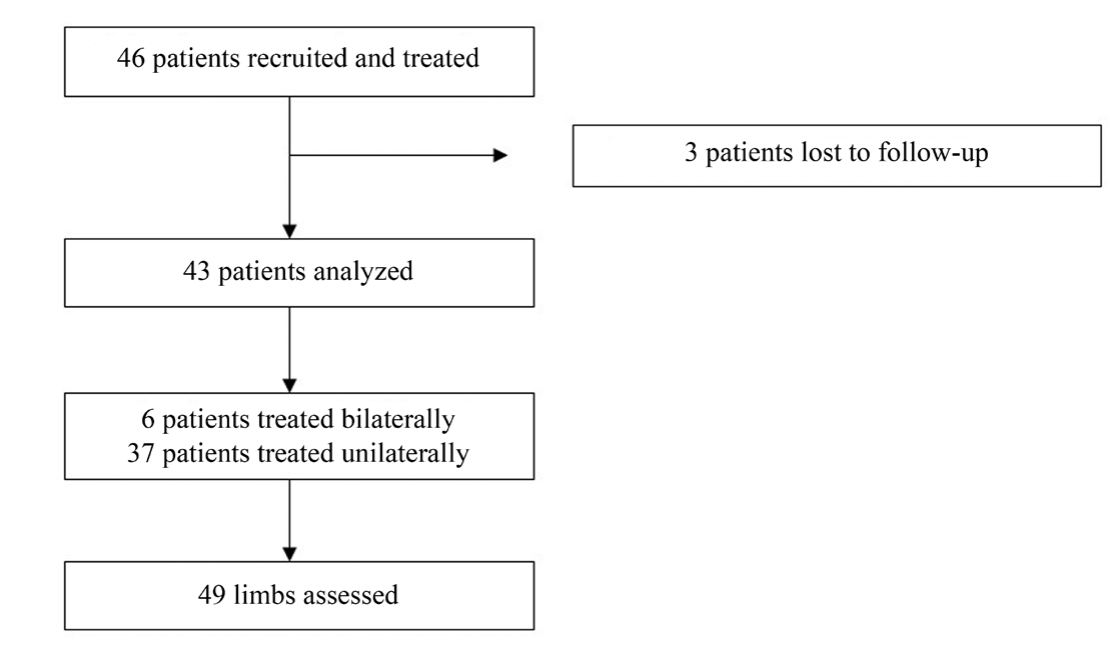
Flow diagram of limbs assessed.

The total number of limbs treated was 52, but 3 (5.7%) patients were lost to follow-up. Thirty-three (76.7%) of the 43 patients analyzed were female and the mean age of the sample was 50.9 years, ranging from 27 to 68 years. Neither side was more prevalent. One patient did not attend the 7-day follow-up consultation and three patients did not attend the 3-month follow-up consultation, but all of the patients analyzed remained in the study up to the 1 year endpoint. Surgery was unilateral in 37 patients and bilateral in 6 patients. The total number of limbs analyzed was therefore 49, as illustrated in Figure 3. The unit of observation analyzed was the limb and when two limbs from the same patient were analyzed, they were treated as independent units.

As can be observed in Table 1, time (in months) had a significant effect on SFJ diameter measurements. This measurement reduced over time, with statistical significance (p < 0.05). In Table 2, it can be observed that the same occurred with stump length, suggesting that it retracts over the study period, also with statistical significance (p < 0.05).

**Table 1:** Changes in diameter of the saphenofemoral junction.

Assessment	n	SFJ diameter (mm)		p
		Mean ± standard deviation	Median (min - max)	
Preoperative	49	10.5 ± 2.6	10.6 (5.1 – 16.3)	
7 days	48	9.4 ± 2	9.5 (5.5 - 14.3)	
3 months	47	7.7 ± 1.6	7.3 (4.4 - 12)	
6 months	49	7.4 ± 1.6	7.2 (4.7 - 12)	
1 year	49	7.4 ± 1.7	7.7 (4.1 - 11.2)	< 0.001

SFJ = saphenofemoral junction; n = number of limbs analyzed.

**Table 2:** Changes in residual stump length.

Assessment	n	Stump length		p
		Mean ± standard deviation	Median (min - max)	
7 days	48	19.1 ± 8	18.2 (6.7 - 40)	
3 months	47	16.8 ± 7.4	16 (3.9 - 31.6)	
6 months	49	16.3 ± 6.6	16 (4 - 30)	
1 year	49	16.3 ± 6.2	15.4 (6.4 - 33.6)	0.002

n = number of limbs analyzed.

**Figure 4:**
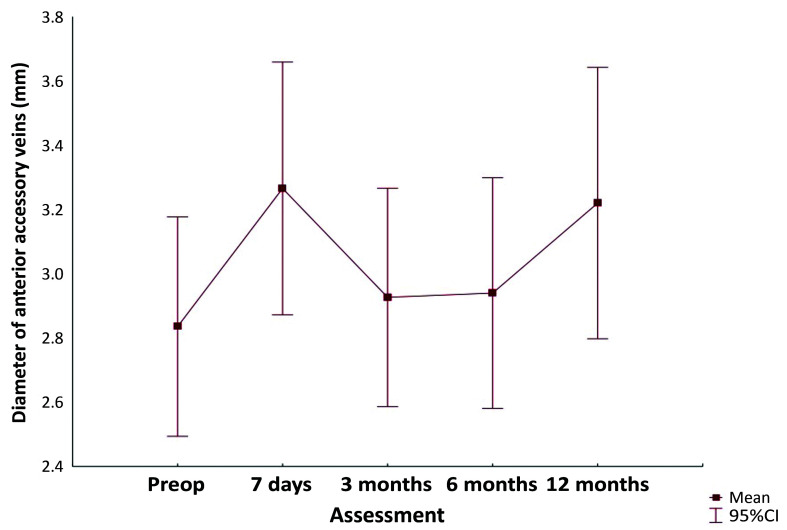
Change in diameter of anterior/posterior accessory veins.

In the total of 49 limbs treated, it was possible to identify 31 anterior/posterior accessory veins with vascular US with Doppler in the preoperative examination and 35 in the 1-year examination. The results indicate that there was no significant effect of time (in months) on the diameter of the anterior accessory vein, with a p value of 0.355. It can be observed in Figure 4 that this measurement remained stable over time.

**Figure 5:**
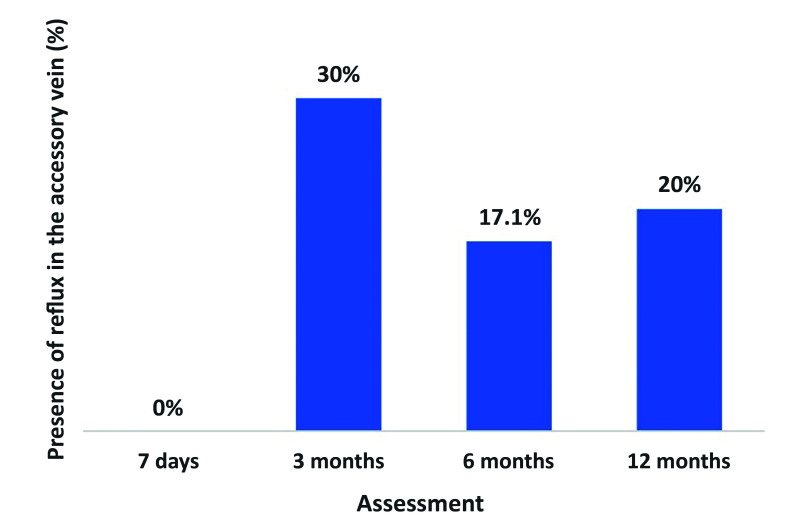
Analysis of development of reflux in the anterior/posterior accessory vein.

Analysis of development of reflux detected no statistical difference when the null hypothesis that the proportion of cases with reflux at 7 days would be equal to the proportion with reflux at subsequent examinations was tested against the alternative hypothesis that these proportions would be different. No significant differences were found between 7 days and 3 months (p = 0.25), 7 days and 6 months (p = 0.06), or 7 days and 1 year (p = 0.06) Therefore, although 20% of the patients did develop reflux in the anterior/posterior accessory saphenous vein over the study period (Figure 5) there was no statistical significance.

**Figure 6:**
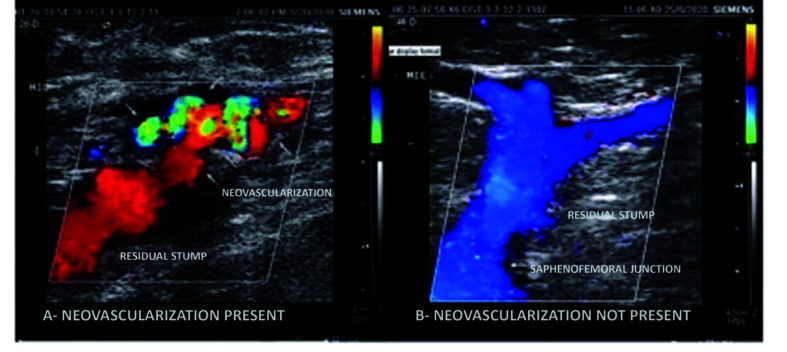
Analysis with color Doppler ultrasonography of the saphenofemoral junction.

Over the course of the 1-year period, development of neovascularization was identified in 7 (14.3%) SFJs. Figure 6 shows a comparison of vascular US with Doppler images from a junction with and a junction without neovascularization.

## DISCUSSION

Surgical treatment is well-established for symptomatic CVI, particularly when associated with GSV reflux.^7^ For many years, conventional surgery was considered the first-choice option for treatment of GSV reflux. It consists of removal of the vein by stripping combined with high ligation at the junction, which is accomplished by ligature of all of its tributaries.^16^^,^^17^

Currently, the leading societies recommend endovenous surgery as first-choice option for treatment of symptomatic GSV reflux, because it is less painful, enables early return to daily activities, and has a lower risk of complications and shorter hospital stays.^16^^-^19 Despite all of these benefits, scientific evidence from well-designed studies and long-term studies proving the superiority of endovenous techniques is still lacking. When compared, both offer similar improvement in quality of life^20^^,^^21^ and both are considered safe and effective.^19^ Long-term recurrence is still questioned, although the results for the two techniques after 3-5 years are similar, particularly when vascular US with Doppler is employed intraoperatively during conventional surgery.^20^^,^^22^

Since endovenous procedures demonstrated good results, Boros et al. conducted a comparative study with patients who underwent the gold standard surgical procedure (endovenous treatment), with or without high ligation of the SFJ, concluding that, in addition to not being indicated, high ligation could lead to higher rates of infection.^23^

A randomized study conducted from 2000 to 2004 by Casoni et al. divided 120 patients into two groups, in which the only difference was whether high ligation of the SFJ was conducted, finding a statistical difference with lower rates of recurrence in the group in which high ligation of the SFJ was not performed, demonstrating its superiority when compared with conventional surgery. Despite these results, the study had a patient sample considered small and the examiners who conducted the imaging exams and clinical examination were not blinded to group membership. Mean time to recurrence of varicose veins was 3.5 years.^14^

Recurrence of varicose veins after surgical procedures is currently one of the greatest challenges faced by vascular surgeons. Inadequate ligature of SFJ tributaries is cited as one of the main causes. Although the anatomy of the SFJ is well-known, there are multiple anatomic variants and this can be a challenge when the conventional technique is chosen, because the classic configuration is only observed in 5.9-15% of patients.^24^^,^^25^

Another challenge related to recurrence rates is understanding whether recurrence is the result of technical failures or neovascularization, or if it may even be part of the natural history of the disease. The cause most often cited as responsible for recurrence after conventional surgery is neovascularization,^18^ but although rates are considerably higher after conventional surgery (21% at 2 years and 27% at 5 years) compared with endovenous surgery (0% at 5 years),^18^ clinical recurrence of varicose veins is not always observed.

Histologically, neovascularization is caused by angiogenesis^10^ and can be identified by formation of new blood vessels in an abnormal position when viewed with vascular US with Doppler.^26^ Hemodynamically, it is interpreted as vascular remodeling, since it is believed that it is formed by dilatation of preexisting small vessels that communicate between the common femoral vein and the GSV and its tributaries. There is increased shear force in the SFJ, which responds with release of endothelial growth factors and nitric oxide, which stimulate dilation.^9^^,^^27^ From 2003 to 2006, Pittaluga et al. conducted 195 great saphenous vein stripping procedures with preservation of the SFJ, and observed a 2-year neovascularization rate of just 1.8%.^13^

In an article published in 2013, Stucker et al. defined three distinct types of reflux at the SFJ: type 1, when there is terminal valve incompetence, pre-terminal competence, and reflux to tributaries, primarily the anterior accessory vein; type 2, when there is terminal valve competence and pre-terminal valve incompetence, suggesting that the reflux drains from the pelvic region; and type 3, when both valves are incompetent.^25^ Ligature of SFJ tributaries in type 2 would therefore be an excessive treatment, since drainage to the common femoral vein would be cut off, increasing the risk of neovascularization. Since one of the exclusion criteria for the present study was reflux in accessory veins, no patients with type 2 reflux were included; although types 1 and 3 were not differentiated.

With the objective of controlling endothelial exposure, interposition of an anatomic barrier at the SFJ using the cribriform fascia or PTFE has proven effective for reducing neovascularization.^7^ However, applying this concept, Heim et al. assessed neovascularization rates after total resection of the residual SFJ stump combined with invagination at the common femoral vein and found 2-year neovascularization rates that were higher than with conventional surgery (20% vs. 9%).^9^ These findings suggest that neovascularization, and consequently recurrence of varicose veins, are not the result of a single causal factor. The study by Heim et al. controlled endothelial exposure at the saphenous stump by invagination. However, conducting this procedure involves increased local manipulation, which could have contributed to the results. In the present study, the option chosen was not to perform ligation of tributaries, with the objective of preserving SFJ hemodynamics, thereby reducing shear forces.

A study conducted by Cappelli et al. with 867 limbs demonstrated that high ligation of the SFJ can increase occurrence of neovascularization, since it changes the SFJ hemodynamics. Preservation of GSV tributaries maintains drainage of the pelvis, thereby reducing vascular remodeling. Preservation of the epigastric vein during thermoablation surgery maintains drainage from the pelvis, facilitating blood flow to the common femoral vein and consequently reducing stasis and inflammation caused by ligation.^11^

When we analyze varicose vein recurrence after minimally invasive surgery, there are two primary causes: recanalization^18^ and insufficiency of the anterior accessory vein.^12^ Considering that we conducted saphenectomy, recanalization was not a possibility. There are three known mechanisms underlying development of reflux in veins feeding the SFJ: true reflux from the SFJ; preexisting reflux not seen on the initial vascular US with Doppler; or reflux hemodynamically masked by the more significant GSV reflux.^12^

In the present study, preoperative examinations identified 31 anterior accessory veins and 35 were identified at the end of the first year. All four of those found after the procedures were already present at the 6-month assessment and just one of them had reflux at 1 year. While the increase in accessory vein diameter over the study period was not statistically significant, (7) 20% of these veins developed reflux, although in 3 (8.57%) of them the reflux was already identified at 3 months, suggesting the possibility of masked reflux, as mentioned above. Although not statistically significant, the number of veins with reflux was considerable and this finding may be related to the short follow-up period.

Change in SFJ diameter was assessed after thermoablation surgery, observing a 72% reduction in diameter after 1 week.^17^ In our study, the junction diameter also changed by 70%, although over a 1 year period, with statistical significance.

The main limitations of this study were presence of just one study group, without comparisons, the low number of limbs assessed, and the short follow-up (12 months), since other studies have suggested that echographic and, in particular, clinical changes can take longer to manifest.^11^

Since the patients were treated on the SUS, it was not possible to make comparisons with endovenous techniques. When performed correctly, conventional surgery does not leave a residual stump. It was therefore not possible to conduct a comparative study of stump behavior between techniques. We understand the complexity of the multiple factors involved in recurrence of varicose veins, some of which are linked to individual factors and not just the surgical technique employed. There is thus no doubt that, in order to recommend a new technique to substitute the well-established conventional saphenectomy, comparative, controlled, and randomized studies are needed, in addition to standardized analyses and definitions of recurrence of neovascularization, which are still lacking in the literature. Notwithstanding, the validity of attempting to do so cannot be denied, considering the need for lower-cost adaptations that can be offered to SUS patients.

## CONCLUSIONS

Twelve months after saphenectomy without high ligation of the SFJ, but with imagination of the residual stump, the stumps had retracted and their diameters had reduced, with no statistically significant transfer of reflux to the anterior accessory veins. The neovascularization rate observed was 14.3%, which is in line with those observed for conventional surgery in the literature, although higher than seen after endovenous methods.

## References

[1] Porciunculla MM, Leiderman DBD, Altenfeder R (2018). Clinical, ultrasonographic and histological findings in varicose vein surgery. Rev Assoc Med Bras.

[2] Oliveira RÁ, Mazzucca A, Pachito D, Riera R, Baptista-Silva J (2018). Evidence for varicose vein treatment : an overview of systematic reviews. Sao Paulo Med J.

[3] Rocha F, Lins E, Almeida C (2020). Quality of life assessment before and after surgery for lower limb varicose veins. J Vasc Bras.

[4] Maffei FH, Magaldi C, Pinho SZ (1986). Varicose veins and chronic venous insufficiency in Brazil: prevalence among 1755 inhabitants of a country town. Int J Epidemiol.

[5] Biemans A, Kockaert M, Akkersdijk G (2013). Comparing endovenous laser ablation, foam sclerotherapy, and conventional surgery for great saphenous varicose veins. J Vasc Surg.

[6] Kemp N (2017). A synopsis of current international guidelines and new modalities for the treatment of varicose veins. Aust Fam Physician.

[7] Wittens C, Davies A, Baekgaard N (2015). Editor’s Choice – Management of chronic venous disease: clinical practice guidelines of the European Society for Vascular Surgery (ESVS). Eur J Vasc Endovasc Surg.

[8] Argyriou C, Papasideris C, Antoniou GA (2018). The effectiveness of various interventions versus standard stripping in patients with varicose veins in terms of quality of life. Phlebology.

[9] Heim D, Negri M, Schlegel U, De Maeseneer M (2008). Resecting the great saphenous stump with endothelial inversion decreases neither neovascularization nor thigh varicosity recurrence. J Vasc Surg.

[10] Theivacumar NS, Darwood R, Gough M (2009). Neovascularisation and recurrence 2 years after varicose vein treatment for sapheno-femoral and great saphenous vein reflux: a comparison of surgery and endovenous laser ablation. Eur J Vasc Endovasc Surg.

[11] Cappelli M, Molino-Lova R, Giangrandi I, Ermini S, Gianesini S (2018). Ligation of the saphenofemoral junction tributaries as risk factor for groin recurrence. J Vasc Surg Venous Lymphat Disord.

[12] Anwar MA, Idrees M, Aswini M, Theivacumar N (2019). Fate of the tributaries of sapheno femoral junction following endovenous thermal ablation of incompetent axial vein – A review article. Phlebology.

[13] Pittaluga P, Chastanet S, Guex J (2008). Great saphenous vein stripping with preservation of sapheno-femoral confluence: hemodynamic and clinical results. J Vasc Surg.

[14] Casoni P, Lefebvre-Vilardebo M, Villa F, Corona P (2013). Great saphenous vein surgery without high ligation of the saphenofemoral junction. J Vasc Surg.

[15] Brasil (2021). Banco de dados do Sistema Único de Saúde-DATASUS.

[16] Lynch P, Clarke M, Fulton G (2015). Surgical management of great saphenous vein varicose veins: a meta-analysis. Vascular.

[17] Casana R, Tolva V, Odero A, Malloggi C, Parati G (2018). Three-year follow-up and quality of life of endovenous radiofrequency ablation of the great saphenous vein with the ClosureFast^TM^ procedure: influence of BMI and CEAP class. Vascular.

[18] Wallace T, El-Sheikha J, Nandhra S (2018). Long-term outcomes of endovenous laser ablation and conventional surgery for great saphenous varicose veins. Br J Surg.

[19] Hassanin A, Aherne T, Greene G (2019). A systematic review and meta-analysis of comparative studies comparing nonthermal versus thermal endovenous ablation in superficial venous incompetence. J Vasc Surg Venous Lymphat Disord.

[20] Sincos IR, Baptista APW, Coelho F (2019). Prospective randomized trial comparing radiofrequency ablation and complete saphenous vein stripping in patients with mild to moderate chronic venous disease with a 3-year follow-up. Einstein (Sao Paulo).

[21] Brittenden J, Cooper D, Dimitrova M (2019). Five-year outcomes of a randomized trial of treatments for varicose veins. N Engl J Med.

[22] Toniolo J, Chiang N, Munteanu D, Russell A, Hao H, Chuen J (2018). Vein diameter is a predictive factor for recanalization in treatment with ultrasound-guided foam sclerotherapy. J Vasc Surg Venous Lymphat Disord.

[23] Boros MJ, O’Brien S, McLaren J, Collins J (2008). High ligation of the saphenofemoral junction in endovenous obliteration of varicose veins. Vasc Endovascular Surg.

[24] Cirocchi R, Henry B, Rambotti M (2019). Systematic review and meta-analysis of the anatomic variants of the saphenofemoral junction. J Vasc Surg Venous Lymphat Disord.

[25] Stücker M, Moritz R, Altmeyer P, Reich-Schupke S (2013). New concept: different types of insufficiency of the saphenofemoral junction identified by duplex as a chance for a more differentiated therapy of the great saphenous vein. Phlebology.

[26] De Maeseneer M, Pichot O, Cavezzi A (2011). Duplex ultrasound investigation of the veins of the lower limbs after treatment for varicose veins - UIP consensus document. Eur J Vasc Endovasc Surg.

[27] Recek C (2015). Significance of reflux abolition at the saphenofemoral junction in connection with stripping and ablative methods. Int J Angiol.

