# Supplementary material for: Assessment of residual stumps 12 months after saphenectomy without high ligation of the saphenofemoral junction
Source: J Vasc Bras. 2021 Jul 5;20:e20210029. doi: 10.1590/1677-5449.210029 (PMC8256878; doi:10.1590/1677-5449.210029)
Supplement: Translation

# Translation: Avaliação do coto residual após 12 meses de safenectomia sem ligadura alta da junção safeno-femoral

**Authors:** Giovanna Golin Guarinello, Francisco Eduardo Coral, Jorge Rufino Ribas Timi, Sarah Folly Machado

**Affiliations:** 1 Hospital Santa Casa de Curitiba – HSCMC, Curitiba, PR, Brasil.; 2 Pontifícia Universidade Católica do Paraná – PUCPR, Curitiba, PR, Brasil.; 3 Universidade Federal do Paraná – UFPR, Curitiba, PR, Brasil.

**Keywords:** varizes, insuficiência venosa, veia safena

## Abstract

**Contexto:**

Atualmente, recomenda-se como primeira opção cirúrgica de varizes a termoablação da veia safena; porém, esse procedimento não é realizado pelo Sistema Único de Saúde do Brasil. Como forma de incluir melhores resultados, técnicas cirúrgicas esforçam-se para mimetizar as novas tecnologias sem seus custos, sendo a principal delas a realização da safenectomia convencional sem ligadura das suas tributárias.

**Objetivos:**

Avaliar a evolução do coto residual após safenectomia sem ligadura alta da junção safeno-femoral associada à invaginação do mesmo, assim como avaliar o comportamento das veias acessórias anterior/posterior.

**Métodos:**

Estudo prospectivo e intervencionista. Foram operados 52 membros pela técnica de safenectomia sem ligadura alta da junção safeno-femoral seguida da invaginação do coto residual. Os pacientes foram avaliados no pré e pós-operatório (7 dias, 3, 6 e 12 meses) através de ultrassonografia vascular com Doppler para análise de diâmetro e extensão do coto residual, diâmetro e refluxo na veia acessória anterior/posterior e presença de neovascularização. A análise estatística foi realizada por média, desvio padrão, mediana, valor mínimo e máximo, frequências e percentuais, teste de Fisher e bimodal.

**Resultados:**

Evidenciou-se um efeito significativo do tempo sobre a medida de diâmetro (p < 0,001) e da extensão (p = 0,002) do coto residual, porém o mesmo não foi observado quanto ao diâmetro (p = 0,355) ou refluxo na veia acessória anterior. Foi identificada neovascularização em 7 (14,3%) membros.

**Conclusões:**

Após a utilização da técnica descrita, o coto residual apresentou retração e diminuição do seu diâmetro no período de 1 ano e não transmitiu refluxo para veia acessória. As taxas de neovascularização foram condizentes com a literatura.

## INTRODUÇÃO

As varizes de membros inferiores afetam cerca de 35-50% da população brasileira^1^^-^4 e são um motivo comum para a procura de atendimento médico. Apesar de sua natureza benigna, apresentam importante impacto na qualidade de vida e na área socioeconômica por despesas com cuidados de saúde e dias de trabalho perdidos^3^^,^^5^^,^^6^.

Entre 60 a 80% dos pacientes com varizes apresentam refluxo na junção safeno-femoral (JSF)^1^, sendo que *o guideline* de insuficiência venosa crônica (IVC) da Sociedade Europeia de Cirurgia Vascular (SECV) recomenda o tratamento cirúrgico ao invés do conservador para varizes não complicadas^7^. O padrão-ouro para a cirurgia da IVC sintomática com insuficiência da veia safena magna (VSM) foi por muitos anos a cirurgia convencional realizada com a ligadura alta das tributárias da JSF, seguida da retirada da VSM por eversão^6^^,^^8^. No entanto, estudos sugerem taxas de recorrência de 25-50% em 5 anos^9^.

Uma das principais causas citadas para tal recorrência é a neovascularização que ocorre através do surgimento de novas veias dilatadas e tortuosas no local de manipulação da JSF^6^^,^^10^^,^^11^. Uma das hipóteses para tal surgimento é a exposição endotelial do coto residual e uma alternativa para diminuição da mesma é a invaginação com fios não absorvíveis ou por meio da interposição de uma barreira anatômica, seja com uso da fáscia cribiforme ou cobertura de politetrafluoretileno (PTFE)^7^.

As terapias endovenosas, atualmente consideradas como primeira escolha para tratamento do refluxo de VSM^7^ segundo o *guideline* da SECV, questionaram o princípio da ligadura de todos os afluentes da JSF, mantendo a mesma no local acima da válvula pré-ostial^11^^-^14. Os resultados dessas técnicas demostraram que a frequência de refluxo da JSF a médio prazo não excede 15%, com drenagem anterógrada das tributárias em direção à veia femoral em 85 a 100% dos casos^13^. Apesar da cirurgia convencional e o tratamento endovenoso apresentarem taxas de recorrência de varizes similares após 2 anos, a presença de neovascularização na JSF aparenta ser mais comum nos pacientes que realizaram a cirurgia convencional^10^. Sendo assim, as técnicas de tratamento endovenoso são realizadas sem ligadura alta da JSF, o que pode ser uma vantagem já que diminuem os índices de neovascularização, uma vez em que não há exposição endotelial, e mantêm a drenagem anterógrada das tributárias em direção à veia femoral.

Apesar das técnicas endovenosas serem menos invasivas e com menores taxas de neovascularização, a cirurgia convencional com ligadura alta do JSF ainda é amplamente realizada^8^^,^^14^. No Brasil, foram realizadas em média 65.728 cirurgias de varizes/ano, considerando cirurgias uni e bilaterais realizadas pelo Sistema Único de Saúde (SUS) entre 2016 e 2019^15^. Considerando que o SUS, assim como os planos de saúde privados, não apresenta cobertura para as técnicas endovenosas, a cirurgia convencional é a modalidade cirúrgica mais realizada no país. Tais dados justificam a pesquisa, de forma a aprimorar as técnicas de menor custo baseadas nos princípios hemodinâmicos aplicados nas técnicas endovenosas.

A manutenção da JSF pode ser preferível à cirurgia convencional porque é menos invasiva e está associada a um risco reduzido de reações inflamatórias locais. A utilização de uma abordagem com menor manipulação, sem dissecar a JSF, parece diminuir o estímulo à neovascularização, assim como manter a drenagem venosa inguinal^13^. As vantagens da cirurgia da VSM sem ligadura alta incluem custos mais baixos do procedimento^8^ em relação às cirurgias endovenosas, cobertura pelos planos de saúde e pelo SUS, uma vez que são utilizados os mesmos materiais da técnica com ligadura alta, e possíveis menores taxas de recidiva em relação à cirurgia convencional^11^. A invaginação do coto residual, associada à técnica acima, busca minimizar ainda mais os riscos de neovascularização.

## OBJETIVOS

Avaliar a evolução do coto residual em cirurgias de safenectomia magna sem ligadura alta da JSF associada à invaginação do coto residual, assim como avaliar o comportamento das veias acessórias anterior/posterior.

## MÉTODOS

Este é um estudo prospectivo e intervencionista, aprovado pelo Comitê de Ética e Pesquisa sob o CAAE: 79980117.1.0000.0020, parecer número 2.824.708. Foram avaliados 46 pacientes submetidos ao tratamento cirúrgico de varizes de membros inferiores a partir da técnica descrita a seguir e por uma mesma equipe de cirurgiões. Os pacientes foram previamente selecionados no ambulatório de cirurgia vascular do SUS após a indicação cirúrgica e dos critérios de inclusão e exclusão. Os pacientes foram recrutados por amostragem consecutiva entre janeiro de 2018 e julho de 2019.

Os critérios de inclusão foram: maiores de 18 anos, presença de refluxo na JSF [> 0,5 segundos na ultrassonografia (US) vascular] e assinatura do termo de consentimento livre e esclarecido. Os critérios de exclusão foram: refluxo na safena acessória anterior e/ou posterior, índice de massa corporal > 35 e história prévia de tratamento da JSF do mesmo membro.

Os pacientes selecionados foram avaliados no pré-operatório através de anamnese, exame físico e US vascular com Doppler do membro a ser operado. Com o intuito de confirmar a presença de refluxo na JSF, descartar a presença do mesmo na veia safena acessória anterior/posterior e medir o diâmetro da junção e da(s) veia(s) acessória(s), novo exame de US vascular com Doppler foi realizado na sala de cirurgia, com paciente em ortostatismo, previamente ao início da anestesia por médico com área de atuação em US vascular.

As avaliações pós-operatórias foram realizadas após 7 dias, 3 meses, 6 meses e 1 ano, quando foram avaliados com US vascular com Doppler (modelo M5 – Mindray) os seguintes parâmetros: diâmetro e extensão do coto residual; presença de refluxo na veia safena acessória anterior/posterior; e presença de neovascularização. Todos os exames de imagem do pós-operatório foram realizados em ortostatismo e pelos mesmos avaliadores do pré-operatório. Foi considerada neovascularização a presença de novas veias tortuosas e com refluxo próximas à JSF. A Figura 1 simplifica a técnica utilizada e faz a comparação da mesma com a cirurgia convencional e a endovenosa.

**Figura 1:** Comparação entre técnicas cirúrgicas - tratamento veia safena magna. (A) Crossectomia; (B) Tratamento endovenoso; (C) Sem ligadura alta + invaginação coto.

Iniciou-se as cirurgias através de uma incisão de aproximadamente 3 cm na prega inguinal do membro acometido. Por meio de mínima manipulação local, foram localizadas a VSM e sua tributária mais distal. Após reparo da mesma abaixo da tributária, com auxílio de duas pinças Kelly, foi realizada a secção da veia seguida de sua ligadura proximal com Vicryl 3-0®, sendo possível evidenciar a exposição endotelial do coto residual. Seguiu-se com a invaginação do coto através de pontos contínuos com fio Mononylon 5-0®. Safenectomia total ou parcial foram realizadas conforme a extensão do refluxo e através da técnica de eversão. Demais ramos varicosos foram tratados através de fleboextração. Após revisão da hemostasia, a pele foi aproximada com Mononylon 5-0®. A Figura 2 mostra a invaginação do coto residual.

**Figura 2:** Técnica cirúrgica - invaginação coto residual. (A) Exposição do endotélio; (B) Invaginação coto.

Quanto à análise estatística, os resultados de variáveis quantitativas foram descritos por média, desvio padrão, mediana, valor mínimo e máximo. Variáveis categóricas foram descritas por frequências e percentuais, e para a associação entre duas variáveis categóricas utilizou-se o teste exato de Fisher.

O cálculo do tamanho da amostra foi realizado com base no resultado dos 15 primeiros membros analisados no estudo, considerando o nível de significância de 5%, poder do teste de 90% e desvio padrão da diferença entre duas avaliações igual a 2. Baseado nesse cálculo seriam necessários 44 membros para detectar com significância a diferença de 1 mm entre duas avaliações. O número foi aumentado em seis unidades para suportar possíveis perdas de seguimento. Sendo assim, o tamanho da amostra foi definido em 50 membros.

Na avaliação do comportamento do coto residual optou-se por analisar ao longo do tempo o diâmetro e a extensão da JSF. Para cada uma das medidas testou-se a hipótese nula de que não há efeito do tempo sobre a medida *versus* a hipótese alternativa de que há. Considerou-se que ao não realizar a ligadura alta da JSF o coto residual poderia transmitir refluxo para as tributárias e causar maior recorrência das varizes. Sendo assim, a veia acessória anterior/posterior foi avaliada, também através do teste de hipótese nula, quanto ao desenvolvimento de refluxo e acompanhamento de seu diâmetro.

Para avaliar o efeito do tempo sobre as medidas de diâmetro da JSF, extensão do coto e diâmetro da acessória foi usado o modelo de efeitos mistos, considerando-se intercepto e inclinação como efeitos aleatórios e tempo como efeito fixo. Para a comparação dos momentos de avaliação da proporção de veias acessórias com presença de refluxo foi usado o teste binomial. Valores de p < 0,05 indicaram significância estatística. Os dados foram analisados por meio do programa computacional IBM SPSS *Statistics* v.20.0. Armonk, NY: IBM Corp.

## RESULTADOS

O número total de membros tratados foi de 52; porém, 3 (5,7%) pacientes perderam o acompanhamento. Dos 43 pacientes analisados, 33 (76,7%) eram do sexo feminino, sendo a média de idade de 50,9 anos, variando de 27 a 68 anos. Não houve prevalência de lateralidade. Um paciente não compareceu à consulta de retorno de 7 dias e três pacientes não compareceram à consulta de retorno de 3 meses; no entanto, todos os pacientes analisados concluíram a pesquisa após 1 ano. Em 37 pacientes a cirurgia foi unilateral e em 6 pacientes a cirurgia foi bilateral. Sendo assim, o número de membros analisados foi de 49, conforme demonstrado na Figura 3. A unidade de observação da análise foi o membro e assumimos que membros do mesmo paciente eram unidades independentes.

**Figura 3:** Fluxograma dos membros avaliados.

Na Tabela 1 podemos observar que há um efeito significativo do tempo (em meses) sobre a medida de diâmetro da JSF. Observa-se que essa medida diminui com o decorrer do tempo e apresenta significância estatística (p < 0,05). Na Tabela 2 podemos observar que o mesmo acontece com a extensão do coto, sugerindo que há uma retração do mesmo ao longo do período, também com significância estatística (p < 0,05).

**Tabela 1:** Evolução do diâmetro da junção safeno-femoral.

Avaliação	n	Diâmetro JSF (mm)		p
		Média ± desvio padrão	Mediana (min - max)	
Pré	49	10,5 ± 2,6	10,6 (5,1 – 16,3)	
7 dias	48	9,4 ± 2	9,5 (5,5 - 14,3)	
3 meses	47	7,7 ± 1,6	7,3 (4,4 - 12)	
6 meses	49	7,4 ± 1,6	7,2 (4,7 - 12)	
1 ano	49	7,4 ± 1,7	7,7 (4,1 - 11,2)	< 0,001

JSF = junção safeno-femoral; n= número de membros avaliados.

**Tabela 2:** Evolução da extensão do coto residual.

Avaliação	n	Extensão do coto		p
		Média ± desvio padrão	Mediana (min - max)	
7 dias	48	19,1 ± 8	18,2 (6,7 - 40)	
3 meses	47	16,8 ± 7,4	16 (3,9 - 31,6)	
6 meses	49	16,3 ± 6,6	16 (4 - 30)	
1 ano	49	16,3 ± 6,2	15,4 (6,4 - 33,6)	0,002

n = número de membros avaliados.

Do total de 49 veias tratadas, foi possível identificar por meio de US vascular com Doppler a presença de 31 veias acessórias anteriores/posteriores no exame pré-operatório e 35 no exame de 1 ano. O resultado indica não haver efeito significativo do tempo (em meses) sobre a medida do diâmetro da acessória anterior, sendo o valor de p = 0,355. Observa-se na Figura 4 que essa medida se mantém estável ao longo do tempo.

**Figura 4:** Evolução do diâmetro da veia acessória anterior/posterior.

Ao avaliar o desenvolvimento de refluxo, não houve diferença estatística quando se testou a hipótese nula de que a proporção de casos com refluxo em 7 dias é igual à proporção de refluxo na avaliação subsequente *versus* a hipótese alternativa de que as proporções são diferentes. Não foi encontrada diferença significativa entre 7 dias e 3 meses (p = 0,25), entre 7 dias e 6 meses (p = 0,06) e entre 7 dias e 1 ano (p = 0,06). Sendo assim, apesar de 20% dos pacientes desenvolverem refluxo na veia safena acessória anterior/posterior ao longo do tempo (Figura 5) não houve significância estatística.

**Figura 5:** Avaliação do desenvolvimento de refluxo na veia acessória anterior/posterior.

No período de 1 ano, foi identificado o desenvolvimento de neovascularização em 7 (14,3%) JSFs. A Figura 6 demonstra a comparação da US vascular com Doppler de uma junção com e sem neovascularização.

**Figura 6:** Análise com eco-Doppler colorido da junção safeno-femoral.

## DISCUSSÃO

A realização de tratamento cirúrgico para IVC sintomática, principalmente quando associada a refluxo da VSM, é bem estabelecida^7^. A cirurgia convencional foi, por muitos anos, considerada como a primeira opção para o tratamento do refluxo da VSM. Consiste na retirada da mesma por eversão associada com a ligadura alta de sua junção, realizada através da ligadura de todas as suas tributárias^16^^,^^17^.

Atualmente, as principais sociedades recomendam a cirurgia endovenosa como primeira opção para o tratamento de refluxo da VSM sintomática, pois acarreta menos dor, permite retorno precoce às atividades diárias, apresenta menor risco de complicações e menor tempo de internamento^16^^-^19. Apesar de todos os benefícios citados acima, ainda não há evidências científicas de estudos bem delineados e de longo prazo que comprovem a real superioridade das técnicas endovenosas. Quando comparadas, ambas proporcionam semelhante melhora da qualidade de vida^20^^,^^21^ e são consideradas seguras e efetivas^19^. Ainda se questiona sua recorrência a longo prazo, com semelhança desse dado entre as técnicas após 3-5 anos, principalmente quando utilizado a US vascular com Doppler no intraoperatório das cirurgias convencionais^20^^,^^22^.

Uma vez que os procedimentos endovenosos mostraram bons resultados, Boros et al. realizaram estudo comparativo com pacientes submetidos ao procedimento cirúrgico padrão-ouro (tratamento endovenoso), realizando ou não a ligadura alta da JSF, e concluíram que, além de não ser indicada, a mesma pode acarretar maiores taxas de infecção^23^.

Estudo randomizado realizado por Casoni et al., no período de 2000 a 2004, com 120 pacientes divididos em dois grupos, sendo sua única diferença a realização ou não da ligadura alta da JSF, constatou diferença estatística com menores taxas de recorrências no grupo em que não foi realizada a ligadura alta da JSF, demonstrando sua superioridade quando comparada com a cirurgia convencional. Apesar dos resultados, o estudo apresentou uma casuística considerada pequena e os examinadores que realizaram o exame de imagem e o exame clínico não foram cegos quanto aos grupos. A recorrência das varizes demorou em média 3,5 anos para acontecer^14^.

A recorrência das varizes após procedimentos cirúrgicos é hoje um dos maiores desafios do cirurgião vascular. Cita-se a ligadura inadequada das tributárias da JSF como uma das principais causas. Apesar de bem conhecida, a anatomia da JSF apresenta múltiplas variações anatômicas e pode ser um desafio quando se opta pela técnica convencional, pois sua conformação clássica é observada em apenas 5,9-15% dos pacientes^24^^,^^25^.

Outro desafio quanto às taxas de recorrência é entender se a mesma resultou de falhas na técnica no aparecimento de neovascularização ou até mesmo como evolução natural da doença. A causa mais citada como responsável por recorrências nas cirurgias convencionais é a neovascularização^18^, mas, apesar de apresentar taxas consideravelmente maiores na cirurgia convencional (21% em 2 anos e 27% em 5 anos) comparada com a cirurgia endovenosa (0% em 5 anos)^18^, a recorrência clínica das varizes não é sempre visualizada.

Histologicamente, a neovascularização decorre de angiogênese^10^ e é identificada pela formação de novos vasos sanguíneos em uma posição anormal na visualização com US vascular com Doppler^26^. Hemodinamicamente, ela é interpretada como um remodelamento vascular, pois acredita-se ser formada a partir da dilatação de pequenos vasos pré-existentes que comunicam a veia femoral comum com a VSM e suas tributárias. Uma vez que há aumento da força de cisalhamento na JSF, a mesma libera fatores de crescimento endotelial e óxido nítrico que estimulariam tais dilatações^9^^,^^27^. Pittaluga et al., no período de 2003-2006, realizaram 195 safenectomias magnas por eversão com preservação da JSF, sendo a taxa de neovascularização após 2 anos de apenas 1,8%^13^.

Em artigo publicado em 2013, Stucker et al. caracterizaram três tipos distintos de refluxo da JSF: tipo 1, quando há incompetência da válvula terminal, porém competência da pré-terminal, escoando refluxo para tributárias, principalmente para veia acessória anterior; tipo 2, quando há competência da válvula terminal e incompetência da pré-terminal, sugerindo que esse refluxo drena da região pélvica; e o tipo 3, quando há insuficiência de ambas as válvulas^25^. A ligadura das tributárias da JSF no tipo 2 seria um tratamento excessivo, uma vez que essa drenagem para veia femoral comum estaria interrompida, aumentando o risco de neovascularização. Como no presente estudo um dos critérios de exclusão foi o refluxo das veias acessórias, não foram incluídos os pacientes com refluxo tipo 2; porém, não foi realizada a diferenciação entre os tipos 1 e 3.

Com o intuito de controlar a exposição endotelial, a interposição de uma barreira anatômica na JSF mostrou-se efetiva em diminuir a neovascularização com uso da fáscia cribiforme e interposição de PTFE^7^. No entanto, Heim et al., utilizando esse conceito, avaliaram as taxas de neovascularização após a realização da ressecção total do coto residual da JSF associada à sua invaginação na veia femoral comum e encontraram taxas de neovascularização após 2 anos maiores que na cirurgia convencional (20% *versus* 9%)^9^. Ambos os achados sugerem que a neovascularização, e consequentemente a recorrência das varizes, não apresenta apenas um fator causal. O estudo de Heim et al. controlou a exposição endotelial no coto da safena através de sua invaginação. Porém, ao fazê-lo houve maior manipulação local, o que pode ter contribuído para tais resultados. No presente estudo, optou-se por não realizar a ligadura das tributárias com o intuito de manter a hemodinâmica da JSF, diminuindo assim as forças de cisalhamento.

Estudo conduzido por Cappelli et al. em 867 membros demonstrou que a ligadura alta da JSF pode aumentar a presença de neovascularização, uma vez que altera a hemodinâmica da mesma. A preservação das tributárias da VSM mantém a drenagem da pelve, diminuindo assim o remodelamento vascular. A preservação da veia epigástrica nas cirurgias de termoablação mantém a drenagem da pelve, facilitando o fluxo de sangue para a veia femoral comum e consequentemente diminuindo a estase e a inflamação causada por sua ligadura^11^.

Quando avaliamos a recorrência das varizes em cirurgias minimamente invasivas, duas são suas principais causas: recanalização^18^ e insuficiência da veia acessória anterior^12^. Visto que neste trabalho realizamos a safenectomia, não há possibilidade de recanalização. São conhecidos três mecanismos para o desenvolvimento do refluxo das veias afluentes da JSF, sendo eles: refluxo verdadeiro proveniente da JSF; refluxo prévio não visualizado na US vascular com Doppler inicial; ou refluxo mascarado hemodinamicamente pelo refluxo de maior importância na VSM^12^.

No presente estudo foram identificadas no pré-operatório 31 veias acessórias anteriores e no final do primeiro ano foram identificadas 35, sendo que todas as quatro evidenciadas posteriormente estavam presentes na avaliação de 6 meses; dessas, apenas uma apresentou refluxo após 1 ano. Apesar de não haver diferença estatística em relação ao aumento do diâmetro da mesma, no decorrer do período avaliado (7) 20% dessas desenvolveram refluxo, porém em 3 (8,57%) o refluxo foi identificado na avaliação de 3 meses, sugerindo um possível refluxo mascarado, como citado anteriormente. Apesar de não apresentar significância estatística, o número de veias com refluxo foi considerável e o achado pode estar relacionado ao curto período de acompanhamento.

A evolução do diâmetro da JSF foi avaliada em cirurgias de termoablação, quando se evidenciou uma diminuição do diâmetro para 72% após 1 semana^17^. Em nosso estudo, o diâmetro da junção apresentou uma mesma relação de 70%, porém no período de 1 ano, com significância estatística.

As principais limitações deste estudo foram a presença de apenas um grupo de estudo, sem comparações, o número reduzido de membros avaliados e o curto período (12 meses) de avaliação, pois outros estudos sugerem que alterações ecográficas e principalmente clínicas podem demorar mais tempo para se manifestar^11^.

Dado que os pacientes operados eram atendidos pelo SUS não foi possível comparar com as técnicas endovenosas. A cirurgia convencional, quando realizada corretamente, não deixa coto residual. Por esse motivo, não foi possível realizar estudo comparativo sobre o comportamento do coto entre essas técnicas. Entendemos a complexidade dos múltiplos fatores envolvidos na recorrência das varizes, sendo alguns relacionados com fatores individuais e não somente com a técnica cirúrgica utilizada. Portanto, não há dúvida que para sugerir uma nova técnica que substitua a consagrada safenectomia convencional são necessários estudos comparativos, controlados e randomizados, assim como padronização nas análises e definições de recorrência e neovascularização ainda não presentes na literatura. Contudo, não se pode negar a validade do mesmo diante da necessidade de adaptações com menores custos para pacientes do SUS.

## CONCLUSÕES

O coto residual após 12 meses de safenectomia sem ligadura alta da JSF associada à invaginação do coto residual apresentou retração e diminuição do seu diâmetro e não transmitiu refluxo estatisticamente significativo para veia acessória anterior. As taxas de neovascularização encontradas foram de 14,3%, condizentes com o encontrado na literatura para cirurgia convencional, porém maiores do que o encontrado nas técnicas endovenosas.
